# Notices

**Published:** 1869-05

**Authors:** 


### NOTICES.

Going Abroad.—Reminiscences of European Travel: 316 pp. 12mo. Published by Hurd & Houghton, Cambridge Riverside Press. This book, by Professor Peabody, of Harvard University, is of more than usual interest; the style, matter and manner are such as to attract the cultivated reader, who, whether he is going abroad, or has been, will find in reading this volume much to instruct, to amuse and to admire. It is the production of a close observer, of a logical mind, and a forcible thinker. He not only goes out of the common route of travel, but, when describing what every traveller sees, he makes observations and brings to view points which most persons overlook; hence every page is instructive, even to old travellers.

Our farmer readers are interested in the following statements made by the Messrs. B. K. Bliss & Son, Seed and Horticulture Warehouse, 41 Park Row and 151 Nassau street, New York. Post office address, Box No. 5712. These gentlemen are known as prompt and reliable business men:

New and Choice Potatoes.—Early Rose.—Among the many thousands of our patrons to whom we furnished this valuable potato last spring, we have yet to hear from the first one who is not fully satisfied with his purchase. The only regret expressed is that they had not procured more. We are daily in receipt of the most flattering testimonials, not only of its earliness and good quality, but of its astonishing productiveness, some of which seem almost fabulous. Several report of having grown *a barrel from a single pound;* a yield of *one hundred fold* is an every-day occurence. One pound, $1; three pounds, $2, by mail, postpaid. One peck (15 pounds), $5; half bushel, $8; one bushel (60 pounds), $15; one barrel (165 pounds), $40. Prices to the trade, in larger quantities, will be given upon application. The freight on all packages by express, boat, or railroad, to be paid by the purchaser. No charge for packages or cartage. *Climax*—a new early variety of great promise. By mail, postpaid, $3 per pound. *Bresee's Prolific*—a new variety, highly recommended for general cultivation; enormously productive; second in quality only to Early Rose. By mail, postpaid, $2 per pound. Upon receipt of $5 we will mail one pound each of the above varieties to any address in the Union. Our descriptive price-list of potatoes sent to all applicants.

American Newspaper Directory, by Geo. P. Rowell & Co., 8vo., 358 pp., $5. The publication of this Directory is a novelty, but one of those "Notions" that are found very useful to the business community.—*Republican, Waverly, Ohio.* Will be the key to fortune for many a stirring business man. It is copious and comprehensive.—*Reporter, Three Rivers, Mich.* It is a great desideratum, and will be the first complete publication of its kind extant. As a reference book it will be invaluable.—*Telegraph, Berlin, Ontario, D. C.* From an inspection we are satisfied that their plan is a good one, and that they are about to produce the most perfect work of the kind ever given to the public.—*Bulletin, Williamsport, Pa.* Seems to have been prepared with honesty as well as industry and intelligence.—*Nation, New York City.* The newspapers are classified, arranged alphabetically by towns, and fully described. This will be a valuable publication for every business man to have.—*Republican, Wooster, O.* It certainly presents a very beautiful typographical appearance, is printed on fine heavy paper, and the various lists, as arranged by States, are compact, neat and convenient for references.—*Star*, *Long Island City*, *N. Y.* The plan of the work is one of great comprehensiveuess and distinctness, and such a classification of newspapers and periodical sheets is presented as will make it almost an indispensable work to those connected with advertising. The work is also most admirably printed.—*Boyd's Shipping Gazette*, *New York City*.

Tribune Essays.—Leading articles contributed to the New York *Tribune*, June 1857 to 1863, by Charles T. Congdon; with an introduction by Horce Greeley. New York: J. S. Redfield, 140 Fulton street. 12mo., 432 pp. Price $2.00. Through the columns of the New York *Tribune* a wide circle of readers is already familiar with some, or all, of the articles of which this volume is composed, and they will, no doubt, be glad to hear of their republication in the present shape. For brilliancy of wit and keenness of satire there has been nothing to compare with them in our day. We notice that the mere announcement of the publication has been warmly and universally welcomed by the press throughout the country, and we predict for the book a large sale.

Wadsworth's Sermons, twenty in number, published by Roman & Co., San Francisco, and 27 Howard street, New York. There is a novelty and a power and a searchingness in both the printed and spoken sermons of this eloquent and distinguished Presbyterian divine, not exceeded by any clergyman we have heard at home or abroad. Price, $2.00. Same by mail. The first two sermons, "God's Thoughts," and "The one Idea," are splendid.

Packard's Monthly, $1 a year, is truly, as its other name imports, "The Young Men's Magazine," and one which all young men can read with profit, and without loss; it richly merits a large circulation.

The London Lancet is issued at the same time in New York and London, monthly, at $5 a year. It is a publication of long standing and high character on both continents among educated physicians, containing, as it does, the latest medical and surgical news of improvement and progress throughout the world. Published at No. 32 Beekman street, by Wm. C. Herald. Postage, 24 cents a year.

How to Read Character is a very beautiful volume, published by Samuel R. Wells, 389 Broadway, New York, being a new and illustrated hand-book of Phrenology and Physiology for students and examiners, with a descriptive chart. To know the character of the persons we deal with is an essential element of success in life, the want of such knowledge has been the pecuniary and moral ruin of millions.

Luscious Ice-Cream is made by Jacob Fussell & Co., 1288 Broadway, New York, and is promptly and faithfully served to families, summer and winter; persons removing to the upper part of the city may feel assured that a better article, at as moderate rates, is not anywhere furnished in New York. All the materials used at this establishment are always pure and fresh, and the best of their kind. Branch offices at 299 Fourth avenue and 190 Chatham square.

